# Circulating growth arrest-specific protein 6 levels are associated with erythropoietin resistance in hemodialysis patients

**DOI:** 10.1186/s40064-016-1681-z

**Published:** 2016-01-13

**Authors:** Miao-Pei Chen, Chien-Wen Chen, Jin-Shuen Chen, Hung-Chung Mao, Chu-Lin Chou

**Affiliations:** Team of Nephrological Research, Ping-Tung Christian Hospital, No. 60, Dalian Rd., Pingtung City, 900 Pingtung County Taiwan, ROC; Hemodialysis Center, Ping-Tung Christian Hospital, Pingtung City, Taiwan, ROC; Division of Nephrology, Department of Internal Medicine, Tri-Service General Hospital, National Defense Medical Center, Taipei, Taiwan, ROC

**Keywords:** Erythropoietin, Growth arrest-specific protein 6, Hemodialysis, High-sensitivity C-reactive protein, Interleukin 6

## Abstract

Growth arrest-specific protein 6 (Gas6) works synergistically with erythropoietin (EPO) to increase the proliferation and maturation of erythroblasts. However, the role of Gas 6 levels on EPO resistance in hemodialysis (HD) patients remains unclear. Therefore, the objective of this study was the first to examine the correlation between plasma Gas6 levels and EPO resistance in HD patients. We enrolled 134 HD patients and 85 healthy individuals. The HD patients were divided into 2 groups: 98 non-EPO-resistant patients and 36 EPO-resistant patients. Plasma levels of Gas6, interleukin 6 (IL-6), high-sensitivity C-reactive protein (hs-CRP), and albumin were quantified. Compared with non-EPO-resistant patients, EPO-resistant patients had elevated plasma concentrations of Gas6 (15.4 ± 3.3 vs. 13.7 ± 3.2 ng/mL, *P* = 0.006), IL-6 (3.1 ± 3.1 vs. 2.1 ± 1.5 pg/mL, *P* = 0.009), and hs-CRP (12.7 ± 25.2 vs. 4.5 ± 5.5 mg/L, *P* = 0.002). In EPO-resistant HD patients, plasma Gas6 levels were negatively correlated with albumin levels (*r* = −0.388, *P* < 0.021). Elevated Gas6 levels are associated with EPO resistance in HD patients. Also, EPO resistance is related to inflammation and malnutrition. Thus, circulating Gas6 levels could be used as the potential marker in HD patients with EPO resistance.

## Background

Erythropoietin (EPO) resistance is one of the most common complications in anemia patients undergoing hemodialysis (HD). Most factors causing EPO resistance are associated with malnutrition, chronic inflammation, hyperparathyroidism, and inadequate dialysis in HD patients, which shorten the lifespan of erythrocytes (Macdougall and Cooper 2002; Kanbay et al. 2010). EPO resistance is a potential risk factor for mortality, cardiovascular disease, and stroke, which are associated with the high-dose use of EPO-stimulating agents and inability to achieve a target hemoglobin level (Kilpatrick et al. 2008; Szczech et al. 2008). Thus, a careful evaluation of EPO resistance is crucial in patient care for improving clinical outcomes.

Growth arrest-specific protein 6 (Gas6), a vitamin K-dependent protein, plays a role in cell growth, cell proliferation, anti-inflammation, and phagocytosis (Tjwa et al. 2008; Ekman et al. 2010a, b). Gas6 is expressed in some cell types, such as endothelial, smooth muscle, and bone marrow cells (Tjwa et al. 2008). It was recently reported that elevated circulating Gas6 levels are correlated with disease activity such as systemic inflammation (Ekman et al. 2010a, b), acute coronary syndrome (Jiang et al. 2009), acute pancreatitis (Uehara et al. 2009), and systemic lupus erythematosus (Ekman et al. 2011). In chronic kidney disease patients, Gas6 levels were associated with renal disease and inversely related to renal function, suggesting that Gas6 levels may serve as a biomarker for disease stage (Lee et al. 2012). In patients with critical limb ischemia, circulating Gas6 levels correlated with survival outcomes, indicating that Gas6 can be considered as a potential biomarker in clinical practice (Ekman et al. 2010a, b).

In anemic mice, Gas6, released by erythroblasts, enhanced and synergized with EPO receptor signaling to facilitate hemoglobin synthesis (Angelillo-Scherrer et al. 2008). However, to date, the correlation between Gas6 and EPO resistance remains unclear in HD patients. Therefore, the objective of this study was the first to investigate the correlation between circulating Gas6 levels and EPO resistance in HD patients.

## Methods

### Subjects

Healthy control and HD subjects were enrolled in this study at the Ping-Tung Christian Hospital. This study was approved by the human research ethics committee of the hospital (Approved No.: IRB171A), and informed consent was obtained from each patient. Between December 2009 and December 2010, 85 healthy subjects were randomly recruited from an unselected population, as the control group. The experimental group included 134 HD patients. Patients with sepsis, iron deficiency, folate deficiency, vitamin B deficiency, bone marrow diseases, and hematopoietic diseases were excluded from this study. The HD patients were further divided into two groups: EPO-resistant patients were defined as those receiving a high dose of EPO ≥ 40,000 U/month for a minimum of 6 months and with hematocrit (Hct) levels <33 %, and non-EPO-resistant patients were defined as those receiving a low dose of EPO < 40,000 U/month for a minimum of 6 months and with Hct levels >33 %. All patients underwent polysulfone HD 3 times/week (dialysis flow of 500 mL/min for 4 h, endotoxin <0.03 EU/mL). After enrollment, fasting morning serum samples were obtained from the subjects and were stored at −80 °C until analysis.

Biochemical assessments were performed, including measurement of Gas6, interleukin 6 (IL-6), high-sensitivity C-reactive protein (hs-CRP), blood urea nitrogen (BUN), albumin, phosphate, calcium, intact parathyroid hormone (i-PTH), Hct, ferritin, serum iron (SI), transferrin saturation (TSAT), total iron-binding capacity (TIBC), red blood cell (RBC) fragility, and Kt/V (product of dialyzer urea clearance and treatment time divided by body urea volume).

#### Measurement of growth arrest-specific protein 6, interleukin 6, and high-sensitivity C-reactive protein

Plasma Gas6 and IL-6 concentrations were determined using commercial enzyme immunoassay kits (R&D Systems, Minneapolis, MN), according to the manufacturer’s instructions (Yanagita et al. 2002; Schuett et al. 2009). Plasma hs-CRP levels were measured using a Beckman Coulter IMMAGE Immunochemistry System (Brea, CA) with a detection limit of 0.2 mg/L (Schuett et al. 2009). The dilution curve was parallel to the standard curve. The inter- and intra-assay coefficients of variation were 8.0–8.7 % and 3.4–7.1 % for Gas6 and 6.5–9.6 and 6.9–7.8 % for IL-6, respectively. Samples were measured in duplicate in each experiment.

#### Measurement of serum biochemical parameters and red blood cell fragility

SI, TSAT, TIBC, BUN, albumin, phosphate, and calcium levels were measured with standard methods using UniCel DxC 800 (Beckman Coulter Inc., USA). Ferritin and i-PTH levels were measured using standard commercial assays (Cobas e411, Roche Diagnostics, USA). Hct levels were measured using standard commercial kits (Sysmex XE-5000, Sysmex Corporation, Japan). TIBC (µmol/L) was calculated as plasma transferrin concentration (g/L) × 1.4, and TSAT was calculated as the ratio of SI to TIBC (SI/TIBC). Urea clearance was expressed as Kt/V (K, dialyzer urea clearance; t, dialysis time; V, body urea volume), which is the measurement of dialysis adequacy. Kt/V values, calculated by Kt/V = Ln (Co/Ct) (Ln, natural logarithm; Co, postdialysis BUN; Ct, predialysis BUN), expressed the changes in plasma BUN levels before and after HD. To evaluate RBC osmotic fragility, 10 concentrations of NaCl, ranging from 0 to 0.9 %, were incubated with 50 µL of RBCs. The absorbance of the supernatant versus water was determined at 575 nm by using a spectrometer (µQuant, BioTek Instruments, Inc.). The NaCl concentration at which 50 % of the RBCs were lysed was considered as the median osmotic fragility (Wu et al. 1998).

### Statistical analysis

Data are expressed as mean ± standard deviation. All statistical analyses were performed using Statistical Package for Social Science (SPSS for Windows, version 19.0; SPSS Inc., Chicago, IL). Statistical differences in variables were compared using the unpaired *t* test. Pearson correlation analysis was used to evaluate the bivariate relationship between individual variables and plasma Gas6 levels. A value of *P* ≤ 0.05 was considered statistically significant.

## Results

### Patient characteristics

The 85 control subjects comprised 33 healthy male and 52 healthy female subjects with an average age of 60.8 ± 10.8 years, and the 134 HD patients comprised 46 male and 88 female patients with an average age of 62.2 ± 13.3 years and an average HD period of 81.0 ± 39.9 months. The HD patients were further divided into 2 groups: (1) 36 EPO-resistant HD patients comprising 11 male and 25 female patients receiving a high dose of EPO ≥ 40,000 U/month for a minimum of 6 months and with Hct levels <33 %; and (2) 98 non-EPO-resistant HD patients comprising 35 male and 63 female patients receiving a low dose of EPO < 40,000 U/month for a minimum of 6 months and with Hct levels >33 %.

### Biochemical characteristics between normal and hemodialysis subjects

The clinical characteristics of the enrolled subjects are shown in Table 1. Compared with healthy subjects, HD patients had significantly higher concentrations of serum ferritin (453.9 ± 325.5 vs. 165.4 ± 110.0 ng/mL, *P* < 0.001), i-PTH (445.7 ± 502.8 vs. 26.4 ± 12.9 pg/mL, *P* < 0.001), phosphate (5.39 ± 1.48 vs. 3.59 ± 0.42 mg/dL, *P* < 0.001), plasma Gas6 (14.16 ± 3.28 vs. 6.65 ± 1.75 ng/mL, *P* < 0.0001), IL-6 (2.35 ± 2.05 vs. 1.15 ± 1.89 pg/mL, *P* < 0.001), and hs-CRP (6.7 ± 14.0 vs. 0.9 ± 0.9 mg/L, *P* = 0.002). In contrast, compared with healthy subjects, HD patients had significantly lower levels of SI (61.2 ± 23.0 vs. 91.3 ± 36.6 μg/dL, *P* < 0.001), TIBC (252.6 ± 59.1 vs. 335.4 ± 62.9 μg/dL, *P* < 0.001), TSAT (25.4 ± 10.9 vs. 29.5 ± 17.3 %, *P* = 0.031), Hct (32.9 ± 2.5 vs. 40.0 ± 3.9 %, *P* < 0.001), and albumin (3.97 ± 0.36 vs. 4.56 ± 0.27 g/dL, *P* < 0.001).

**Table 1:** Clinical characteristics of study subjects

Item	Healthy control	Hemodialysis patient	*P* value
*N*	85	134	
Ferritin (ng/ml)	165.4 ± 110.0	453.9 ± 325.5	<0.001
SI (μg/dl)	91.3 ± 36.6	61.2 ± 23.0	<0.001
TSAT (%)	29.5 ± 17.3	25.4 ± 10.9	0.031
TIBC (μg/dl)	335.4 ± 62.9	252.6 ± 59.1	<0.001
i-PTH (pg/ml)	26.4 ± 12.9	445.7 ± 502.8	<0.001
Hematocrit (%)	40.0 ± 3.9	32.9 ± 2.5	<0.001
Albumin (g/dl)	4.56 ± 0.27	3.97 ± 0.36	<0.001
Phosphate (mg/dl)	3.59 ± 0.42	5.39 ± 1.48	<0.001
Calcium (mg/dl)	9.18 ± 0.37	9.33 ± 0.87	0.136
Gas6 (ng/ml)	6.65 ± 1.75	14.16 ± 3.28	<0.001
IL-6 (pg/ml)	1.15 ± 1.89	2.35 ± 2.05	<0.001
hs-CRP (mg/l)	0.9 ± 0.9	6.7 ± 14.0	0.002
RBC fragility test (% of NaCl)	0.426 ± 0.034	0.414 ± 0.049	0.075

Data are mean ± SD

*SI* serum iron, *TSAT* transferring saturation, *TIBC* total iron-binding capacity, *i-PTH* intact parathyroid hormone, *Gas6* growth arrest-specific protein 6, *IL-6* Interleukin 6, *Hs-CRP* high sensitivity C-reactive protein, *RBC* red blood cell

### Elevated growth arrest-specific protein 6 and inflammation levels in erythropoietin-resistant hemodialysis patients

A comparison of blood biochemistry data between the EPO-resistant and non-EPO-resistant HD patients is presented in Table 2. Compared with non-EPO-resistant patients, EPO-resistant patients had elevated concentrations of plasma Gas6 (15.4 ± 3.3 vs. 13.7 ± 3.2 ng/mL, *P* = 0.006), IL-6 (3.1 ± 3.1 vs. 2.1 ± 1.5 pg/mL, *P* = 0.009), and hs-CRP (12.7 ± 25.2 vs. 4.5 ± 5.5 mg/L, *P* = 0.002). However, non-EPO-resistant patients had higher Hct concentrations than EPO-resistant patients.

**Table 2:** A comparison of blood biochemistry data between the EPO-resistant and non-EPO-resistant HD patients

Item	Non-EPO resistance	EPO resistance	*P* value
*N*	98	36	
Ferritin (ng/ml)	445.4 ± 330.8	472.9 ± 310.0	0.668
SI (μg/dl)	62.6 ± 22.3	57.8 ± 24.8	0.293
TSAT (%)	25.6 ± 11.2	24.5 ± 10.0	0.618
TIBC (μg/dl)	257.7 ± 58.0	241.4 ± 64.0	0.167
i-PTH (pg/ml)	417.7 ± 460.0	514.0 ± 609.0	0.331
Hematocrit (%)	33.5 ± 2.4	31.3 ± 2.7	<0.001
Albumin (g/dl)	4.0 ± 0.3	3.9 ± 0.4	0.173
Phosphate (mg/dl)	5.3 ± 1.5	5.6 ± 1.5	0.233
Calcium (mg/dl)	9.4 ± 0.8	9.2 ± 0.9	0.224
Gas6 (ng/ml)	13.7 ± 3.2	15.4 ± 3.3	0.006
IL-6 (pg/ml)	2.1 ± 1.5	3.1 ± 3.1	0.009
hs-CRP (mg/l)	4.5 ± 5.5	12.7 ± 25.2	0.002
Kt/V	1.79 ± 0.29	1.73 ± 0.32	0.272
RBC fragility test (% of NaCl)	0.413 ± 0.05	0.417 ± 0.04	0.700

Data are mean ± SD

*EPO* erythropoietin, *SI* serum iron, *TSAT* transferring saturation, *TIBC* total iron-binding capacity, *i-PTH* intact parathyroid hormone, *Gas6* growth arrest-specific 6, *IL-6* Interleukin 6, *Hs-CRP* high sensitivity C-reactive protein, *RBC* red blood cell

### Negative correlation between growth arrest-specific protein 6 and albumin levels in erythropoietin-resistant hemodialysis patients

A comparison of clinical variables associated with Gas6 levels is presented in Table 3. In EPO-resistant HD patients, plasma Gas6 levels were negatively correlated with albumin levels (*r* = −0.388, *P* < 0.021). Moreover, in healthy controls, Gas6 levels were positively correlated with IL-6 levels but negatively correlated with Hct and albumin levels.

**Table 3:** A comparison of clinical variables associated with Gas6 levels

Item	Healthy control (*N* = 85)		Non-EPO resistance (*N* = 98)		EPO resistance (*N* = 36)	
Variable	*r*	*P* value	*r*	*P* value	*r*	*P* value
Ferritin	0.022	0.845	−0.140	0.167	−0.206	0.236
SI	−0.192	0.078	−0.060	0.558	−0.148	0.395
TSAT	−0.117	0.287	−0.09	0.373	−0.154	0.378
TIBC	−0.018	0.871	0.067	0.508	0.068	0.699
i-PTH	0.043	0.694	0.139	0.171	0.056	0.748
Hematocrit	−0.248	0.022	0.141	0.164	−0.174	0.318
Albumin	−0.320	0.003	−0.096	0.343	−0.388	0.021
Phosphate	0.034	0.756	−0.116	0.254	−0.172	0.322
Calcium	−0.042	0.704	−0.003	0.977	0.043	0.805
IL-6	0.321	0.003	−0.043	0.675	0.149	0.393
hs-CRP	0.066	0.546	−0.017	0.868	0.013	0.941
Kt/V	–	–	−0.065	0.524	0.300	0.081
EPO dose	–	–	−0.118	0.244	−0.151	0.387

The correlations were estimated using the Pearson correlation test

*Gas6* growth arrest-specific 6, *EPO* erythropoietin, *SI* serum iron, *TSAT* transferring saturation, *TIBC* total iron-binding capacity, *i-PTH* intact parathyroid hormone, *Hct* hematocrit, *IL-6* Interleukin 6, *Hs-CRP* high sensitivity C-reactive protein, *EPO* erythropoietin

## Discussion and conclusions

Our study is the first to demonstrate that EPO-resistant HD patients have higher Gas6 levels than non-EPO-resistant HD patients. Our results showed that elevated circulating Gas6 levels were associated with EPO resistance in HD patients. In addition to Gas6 levels, concentrations of inflammation markers, s uch as IL-6 and hs-CRP, were increased in EPO-resistant HD patients. Further analyses showed that circulating Gas6 levels were negatively correlated with albumin levels in EPO-resistant HD patients. Elevated circulating Gas6 levels may serve as a clinical marker for EPO resistance in HD patients.

In our study, circulating Gas6 levels were elevated in EPO-resistant HD patients. Our data are consistent with the results of recent studies. Studies show that circulating Gas6 levels are associated with disease activity, such as severe sepsis (Borgel et al. 2006) or septic shock (Gibot et al. 2007), acute coronary syndrome (Jiang et al. 2009), and glomerular injury in nephrotoxic nephritis (Yanagita et al. 2002). Moreover, elevated Gas6 levels were associated with increased mortality in patients with severe peripheral arterial disease, characterized by rest pain and gangrene in the legs (Ekman et al. 2010a, b). In chronic kidney disease patients, Gas6 levels increased with kidney disease stage progression and were inversely related to the estimated glomerular filtration rate (*P* < 0.0001). In mouse models, Gas6 enhanced EPO receptor signaling stimulating hemoglobin production, thus playing the role of a hemoglobin regulator (Angelillo-Scherrer et al. 2008). In our study, thus we observed that a clinical marker associated with EPO resistance in HD patients were linked to circulating Gas6 levels.

In addition to circulating Gas6 levels, plasma IL-6 and hs-CRP levels were significantly higher in EPO-resistant patients than in non-EPO-resistant patients. Some other studies have also reported elevated IL-6 and hs-CRP levels in EPO-resistant HD patients (Abe et al. 2011a, b; Shinzato et al. 2008). This indicates that EPO resistance is linked to systemic inflammation in HD patients. Recently, Gas6 was proposed to be a potential regulator of immune response in the development of systemic inflammation (Yanagita et al. 2002; Borgel et al. 2006; Hurtado and de Frutos 2010). In mouse models of inflammation, Gas6 induced the sequestration of circulating platelets and leukocytes on activated endothelium to form circulating leukocyte-platelet conjugates, resulting in thrombosis and inflammation (Tjwa et al. 2008). Moreover, it is known that inflammatory cytokines, such as hs-CRP, IL-6, and tumor necrosis factor-α, suppress erythropoiesis, resulting in inadequate response to recombinant human EPO treatment (Abe et al. 2011a, b; Shinzato et al. 2008; Goicoechea et al. 1998; Kalantar-Zadeh et al. 2004). In our study, Hct levels were significantly lower in EPO-resistant HD patients than in non-EPO-resistant HD patients (31.3 vs. 33.5 %, *P* < 0.001).

Furthermore, plasma Gas6 levels were negatively correlated with serum albumin levels (*r* = −0.388, *P* < 0.021) in EPO-resistant HD patients, implying that EPO resistance could be linked to nutritional status. Recently, Lee et al. (2012) reported that higher Gas6 levels were associated with low albumin levels in chronic kidney disease patients, suggesting a possible connection between malnutrition and inflammation associated with worsening renal function. Moreover, resistance to EPO treatment was associated with low albumin levels in HD patients with depression (Afsar 2013). Furthermore, a lower response to EPO treatment was observed in diabetic HD patients with malnutrition-inflammation-atherosclerosis syndrome (Abe et al. 2011a, b). Overall, EPO resistance is expected to be associated with nutritional and inflammation status (Bamgbola et al. 2009).

RBC fragility could be augmented by inadequate dialysis, which modifies cell membrane structure and shortens RBC lifespan (Stenvinkel and Barany 2002; Sotirakopoulos et al. 2004). In our study, RBC fragility levels and Kt/V values did not significantly differ between EPO-resistant and non-EPO-resistant HD patients, indicating adequate dialysis in both groups. Moreover, serum ferritin levels were adequate and did not significantly differ between the two groups. Sepsis, folate deficiency, vitamin B deficiency, bone marrow diseases, and hematopoietic diseases were not considered as factors causing EPO resistance in HD patients. The exclusion of these factors could have strengthened our study to demonstrate an association between circulating Gas6 levels and EPO resistance in HD patients.

In conclusion, ours is the first study to show elevated circulating Gas6 levels in EPO-resistant HD patients. Compared with non-EPO-resistant HD patients, EPO-resistant HD patients had significantly increased concentrations of plasma Gas6, IL-6, and hs-CRP. Moreover, circulating Gas6 levels were negatively correlated with albumin levels in EPO-resistant HD patients. Thus, EPO resistance in HD patients is associated with inflammation and nutritional status. Circulating Gas6 levels might be used as a potential biomarker for EPO resistance in HD patients. However, the cross-sectional design of our study was a limitation, and therefore, the relevance of these findings needs to be further investigated in longitudinal cohort studies.

## Abbreviations

BUN: blood urea nitrogen
EPO: erythropoietin
Gaas6: growth arrest-specific protein 6
HD: hemodialysis
hs-CRP: high-sensitivity C-reactive protein
Hct: hematocrit
IL-6: interleukin 6
i-PTH: intact parathyroid hormone
RBC: red blood cell
SI: serum iron
TSAT: transferrin saturation
TIBC: total iron-binding capacity
